# Oral Microbiota Development in Early Childhood

**DOI:** 10.1038/s41598-019-54702-0

**Published:** 2019-12-13

**Authors:** Beatrice Kennedy, Sari Peura, Ulf Hammar, Silvia Vicenzi, Anna Hedman, Catarina Almqvist, Ellika Andolf, Göran Pershagen, Johan Dicksved, Stefan Bertilsson, Tove Fall

**Affiliations:** 1 Department of Medical Sciences, Molecular Epidemiology and Science for Life Laboratory, Uppsala University, Uppsala, Sweden; 2 Department of Forest Mycology and Plant Pathology, Science for Life Laboratory, Swedish University of Agricultural Sciences, Uppsala, Sweden; 3 School of Medicine, University of Tasmania, Hobart, Australia; 4 Department of Medical Epidemiology and Biostatistics, Karolinska Institutet, Stockholm, Sweden; 5 Unit of Pediatric Allergy and Pulmonology at Astrid Lindgren Children’s Hospital, Karolinska University Hospital, Stockholm, Sweden; 6 Department of Clinical Sciences, Danderyd Hospital, Stockholm, Sweden; 7 Institute of Environmental Medicine, Karolinska Institutet, Stockholm, Sweden; 8 Department of Animal Nutrition and Management, Swedish University of Agricultural Sciences, Uppsala, Sweden; 9 Department of Ecology and Genetics, Limnology, Uppsala University, Uppsala, Sweden; 10 Department of Aquatic Sciences and Assessment, Swedish University of Agricultural Sciences, Uppsala, Sweden; 11 Centre for Occupational and Environmental Medicine, Region Stockholm, Stockholm, Sweden

**Keywords:** Genetics research, Molecular medicine

## Abstract

Early life determinants of the oral microbiota have not been thoroughly elucidated. We studied the association of birth and early childhood characteristics with oral microbiota composition using 16 S ribosomal RNA (rRNA) gene sequencing in a population-based Swedish cohort of 59 children sampled at 6, 12 and 24 months of age. Repeated-measurement regression models adjusted for potential confounders confirmed and expanded previous knowledge about the profound shift of oral microbiota composition in early life. These alterations included increased alpha diversity, decreased beta diversity and alteration of bacterial composition with changes in relative abundance of 14 of the 20 most common operational taxonomic units (OTUs). We also found that birth characteristics, breastfeeding and antibiotic use were associated with overall phyla distribution and/or with the relative abundance of specific OTUs. Further, we detected a novel link between morning salivary cortisol level, a physiological marker of neuroendocrine activity and stress, and overall phyla distribution as well as with decreased abundance of the most common OTU mapped to the *Streptococcaceae* family. In conclusion, a major part of the maturation of the oral microbiome occurs during the first two years of life, and this development may be influenced by early life circumstances.

## Introduction

The oral microbiota represents a complex, dynamic, and heterogeneous bacterial ecosystem^1^, and its composition has been discussed in relation to oral health^2,3^ and to cardiovascular disease^4^. However, recent studies using 16 S ribosomal RNA (rRNA) sequencing have also linked the oral microbiome in childhood to allergy and asthma diagnoses^5^, weight gain trajectory^6^, and autism spectrum disorder^7^.

The 16 S rRNA amplicon sequencing approach has also been employed to study the neonatal oral microbiota development trajectory, and associations have been noted with antibiotic treatment during pregnancy^8^, gestational age^9,10^, mode of delivery^11–13^ and neonatal diet^14^. Few longitudinal 16 S rRNA sequencing studies have however investigated the oral microbiota development during subsequent early childhood^15,16^. A recent Swedish longitudinal study reported an association between mode of delivery and oral microbial community composition at 3 and 6 months of age^17^, as well as more long-standing associations between partial breastfeeding and early life antibiotic treatment with oral microbiota composition until 7 years of age. However, the participants in that study all had a family history of allergic disease, and a study of potential determinants of oral microbiota development in early childhood in a population-based setting is warranted.

Further, measures of salivary cortisol mirror the biologically active cortisol level in the body, and have been shown to reflect individual stress reactivity and level of acute and/or chronic stress^18^. In smaller cross-sectional studies, salivary cortisol has been associated with the presence of cariogenic bacteria in the oral cavity, as assessed through bacterial cultures, and with the development of dental caries in children^19–21^. No previous study has however investigated the association between salivary cortisol and oral microbiota community development.

In this longitudinal population-based cohort study, we combined results from repeated saliva sampling with prospectively collected information from questionnaires and from national birth and prescription registers. The overall scope of the study was to explore early life determinants for the oral microbiota development trajectory including maternal microbiota, birth characteristics, breastfeeding, antibiotic treatment and exposure to a furry pet. In addition, we obtained the results from repeated analyses of morning salivary cortisol, which uniquely allowed us to investigate the potential association between salivary cortisol and oral microbiota in young children.

## Material and Methods

### The Born into Life cohort

The Born into Life cohort is a prospective longitudinal birth cohort led and maintained by researchers at Karolinska Institutet in Sweden^22^. The main initial scope of the Born into Life cohort was to investigate how maternal, neonatal, and early life circumstances may influence somatic health in childhood and beyond. To this end, women previously enrolled in the LifeGene study^23^, who were living in the Stockholm County Council area and who became pregnant between 2010 and 2012 were invited to participate. Time-points for questionnaires and biomarker sampling were pre-determined before the initiation of the Born into Life cohort. The time-point at gestational week 26–28 was chosen in order to assess women late in pregnancy, but not too close to full term as this would entail a risk of missing women giving pre-term birth. The time-points for the child, at ages 6, 12 and 24 months, were arbitrary time-points chosen to be spread evenly across the first two years of life. There were investigation at other time-points, but salivary samples were not included at those assessments. The maternal cohort comprised of 107 pregnant women, and 93 children were included in the overall Born into Life child cohort at birth. We excluded 28 mothers and 27 children that did not participate in any follow-up after birth.

### Questionnaire data

Mothers responded to questionnaires at inclusion in LifeGene as well as during pregnancy at gestational week 26–28. The mother or father responded to Born into Life questionnaires at child age 6, 12 and 24 months. We extracted information on maternal highest attained education level from the LifeGene questionnaire. We obtained information on number of months of exclusive breastfeeding (categorized as 0–2, 3–4, or 5+) from the Born into Life questionnaire at 6 months. Subsequent information on exclusive breastfeeding from the later questionnaires at 12 and 24 months were not included as the upper age-range of the pre-determined response categories remained 5+ months even at subsequent follow-up. We also included time-updated information on exposure to furry pet, defined as presence of a cat or a dog in the household, from the Born info Life questionnaires at 6, 12 and 24 months.

### National health register data

All residents in Sweden have a unique personal identification number, given at birth or immigration, which enabled record linkages of the mothers and their children to Swedish national health registers. From the Medical Birth Register we obtained information on maternal body mass index (BMI) and maternal smoking in the first trimester, parity (nullipara or multipara), and birth characteristics including the infant’s sex, day of birth, mode of delivery (vaginal or caesarean), gestational age in weeks, and birthweight in grams. Time-updated information on prescription of antibiotics to the child was extracted from the Swedish Prescribed Drug Register. Use of antibiotics was analysed both as time-updated ever-use, and use within the last 90 days at saliva sampling occasion.

### Saliva samples

Evening and morning saliva samples were collected from the children at 6, 12, and 24 months of age, and from the mothers at gestational week 26–28 (for sample instructions see Supplementary Information). Once the samples had been handed in, they were centrifuged for two minutes, transported at standard refrigerator temperature and then stored at −20 °C until cortisol analysis^22^. After cortisol analysis, the remaining sample was stored at −80C.

### Cortisol assessment

Salivary cortisol concentration were analysed at the Centre for Child Research, Stockholm South General Hospital, Stockholm, Sweden, using the standardised CORT-CT2 radioimmunoassay kit (Cisbio Bioassays, Codolet, France) according to the manufacturer’s instructions. The detection interval ranged from concentrations of 1–100 nmol/L. Inter-assay and intra-assay variations were below 5%. Each sample was analysed twice and the mean value of these were used for analysis^22^. Salivary cortisol level was log-transformed in all analyses. As the microbiota analysis was performed in evening saliva samples, we only included morning salivary cortisol as anexposure in our regression analyses, as we wanted to reduce the risk of potential bias from a matrix effect. The saliva matrix refers to the overall saliva composition and all other components of a saliva sample than the analytes of interest, i.e water concentration and pH level, or presence of food or drug residues^24^. Analysing both cortisol and microbiota from the same saliva samples may thereby introduce spurious associations due to sample confounding.

### Microbiota analysis and quality control

Prior to DNA extraction, the analysis order was randomized across samples. Subsequently, DNA from 241 evening saliva samples (156 samples from children, 85 samples from mothers) and 5 negative controls was extracted using MoBio PowerSoil DNA kit (Mobio, Solana beach, CA, USA). After sequencing of the v3 and v4 regions of 16 S rRNA regions (for PCR protocol see Supplementary Information), the samples were purified with AMPure, quantified using Qubit (ThermoFisher Scientific) with dsDNA high-sensitivity assay. 216 barcoded samples with individual dual indices passed this QC step and were pooled in equimolar amounts into two pools of 106 and 110 samples each. The amplicons were sequenced using the Illumina MiSeq technology (2*300 cycles) at Science for Life Laboratory (Uppsala University, Sweden). The resulting reads were processed using Mothur^25^, following the standard operation protocol^26^, except for operational taxonomic unit (OTU) clustering, which was done using Vsearch (abundance-based greedy clustering;^27^) as implemented in Mothur. All samples with less than 10472 sequences were removed (n = 1) and the remaining samples were rarefied to this sequencing depth using Mothur. A cut-off of 97% identity between sequences was used to delineate OTUs. The classification of the operational taxonomic units (OTUs) was done using the Silva database (version 128). In total we detected 24539 OTUs, whereof 5224 in more than one sample. Sequences included in our analyses has been submitted to the Sequence Read Archive (SRA) open repository under reference number PRJNA575550.

### Study population

**Figure 1:**
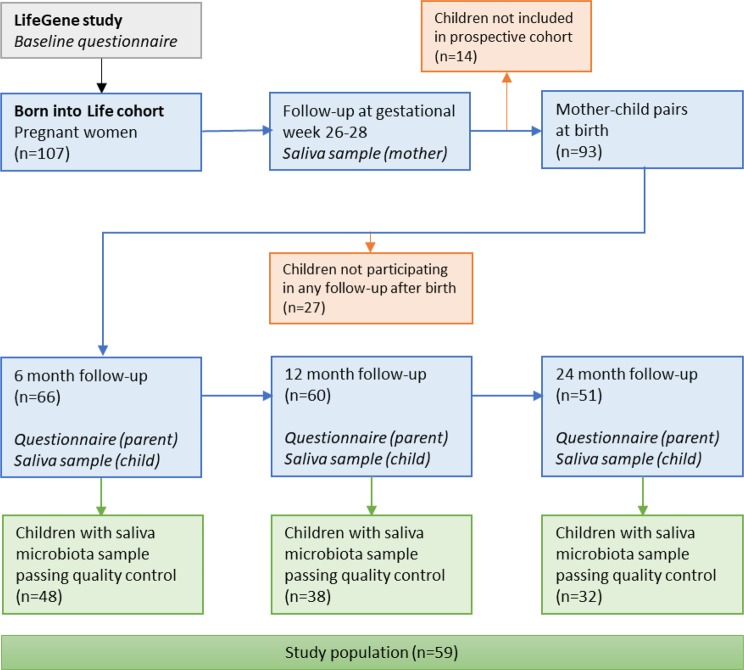
Flowchart of the study population.

Fourteen saliva samples from children had an abnormally low diversity and community composition, possibly implying contamination, and those were accordingly removed from the dataset. After all exclusions, 118 samples from 59 children remained, as well as 55 samples from their mothers. These 59 children constituted our final study population (Fig. 1).

### Statistical analysis

#### Strategy

We applied a series of repeated-measurement regression models to evaluate the association of each of a set of exposures on four different measures of microbiota composition. Since the separate exposure models might require adjustments for different sets of covariates, we created a directed acyclic graph (DAG)^28^ prior to the analysis phase. The DAG is a graphical presentation of the theoretical framework and potential underlying causal structures connecting exposures and covariates with the microbiota outcome in our study (Fig. S1). The hypothesized causal structure is based on previous literature. Using the DAG, we could apply the d-separation criteria and identify the potential confounders for each model (Table 1), as suggested by Greenland *et al*.^29^. The results from all models were then evaluated jointly for statistical significance using a false discovery rate (FDR) of 10%, where FDR was calculated using the Benjamini-Hochberg method^30^. All data was de-identified centrally before data delivery to the researchers and all statistical analyses were performed using Stata v.14 (Stata, College Station, TX, USA).

**Table 1:** Covariates included in the different models.

Exposure	Confounders
Age of child	—
Maternal oral microbiota	Age, maternal age, maternal BMI*
Parity	Age, maternal age.
Gestational age	Age, maternal education level*
Birth weight	Age, maternal BMI, maternal education level, parity, sex, gestational age*
Mode of delivery	Age, maternal BMI, parity, gestational age, birth weight
Exclusive breastfeeding	Age, maternal age, maternal education level, parity, mode of delivery, gestational age, birth weight*
Morning salivary cortisol level	Age, maternal education level, gestational age, birth weight*
Antibiotic treatment (within 90 days or ever)	Age, maternal education level, sex, gestational age
Exposure to furry pet	Age, maternal age, maternal education level

*We identified maternal smoking during pregnancy as a potential confounder. However, in our maternal cohort no women reported smoking whilst pregnant, and this variable is therefore not included in the model.

BMI: Body Mass Index.

#### Models and outcomes

In the main analyses, continuous exposures were modelled using restricted cubic splines with three knots, to account for possible non-linearity in the relationship with the outcomes. Knots were placed a priori according to Harrell’s recommendations^31^.

We considered four different aspects of oral microbiota composition. The association of all exposures with (1) **alpha diversity**, assessed by the inverse Simpson index^32^, was analysed using linear mixed models with restricted maximum likelihood and participant ID as random effect. For (2) overall composition based on **beta diversity**, assessed through non-metric multidimensional scaling (NMDS) calculation^33–36^ using Bray-Curtis distances with the R package vegan based on the rarefied data, we used bivariate linear mixed effects model with participant-ID as a random effect, evaluating both NMDS-axes jointly. This model was fitted by using the generalized structural equation command *gsem* in Stata. The test for association between mother’s beta diversity and child’s beta diversity at 6, 12 and 24 months was performed using multivariate regression, jointly evaluating both NMDS-axes. The outcome (3) **proportion of each bacterial phylum** was analysed using fractional multinomial logistic models with clustered robust standard errors^37^ (*fmlogit*^38^) with participant identity as cluster, which is a way of modelling proportional outcomes. The proportions of each bacterial phyla in the saliva were calculated for phyla present in at least 75% of the samples, and all phyla were evaluated jointly. For the (4) **relative abundance of the 20 most common OTUs** (Table S1), the 20 most common OTUs were selected by rank-transforming OTU abundance within each sample (with absent OTUs given the lowest possible rank), and choosing the 20 OTUs with the highest average rank across all samples. The relative abundance of each OTU was analysed using fractional logistic regression, again with participant identity as cluster. The latter model yielded relative proportion ratios (RPR). An RPR > 1 indicates a higher relative abundance of the OTU in exposed group compared to non-exposed, and RPR < 1 a lower relative abundance.

To specifically evaluate the maternal salivary microbiota composition as an exposure, the maternal and child alpha diversity, beta diversity, proportions of phyla, and 20 most common OTUs were compared, respectively (e.g. maternal alpha diversity was explored in association with child alpha diversity).

#### Sensitivity analyses

We used ordered logistic regression (or, for multivariate outcomes, ordinal generalized structural equations) with cluster-robust standard errors for all exposure-outcome associations as a sensitivity analysis. Ordered logistic regression is a semi-parametric method which generalizes the Mann-Whitney test^31^. Any findings where FDR was below 10% in the main analysis but the sensitivity analysis had a p-value > 0.05 were assumed to be false positive and were not considered or discussed further.

In addition, we also analysed beta diversity using the permutational multivariate ANOVA (PERMANOVA) method^39^, as implemented in the R-package *vegan*. We used the wrapper function adonis_II from the R-package RVaideMemoire^40^ to get type II sum of squares. Independence was assumed, due to individuals having non-equal number of observations. We determined significance using the FDR cut-off from the main analyses, corresponding to a p-value of 0.0209. These results are shown in the Supplementary material (Table S4).

#### Post-hoc tests

For alpha diversity as well as for the 20 most common OTU:s we added an interaction term between exposure and age to all models with a exposure-outcome association passing the significance thresholds. If the p-value for this interaction term was below 0.05, interaction was deemed present. If an exposure was associated with distribution of bacterial phyla proportions, we carried out post-hoc tests, testing all phyla present in more than 75% of the samples individually using fractional logistic regression with cluster-robust standard errors. These post-hoc tests were not corrected for multiple comparisons. Associations with a p-value <0.05 in both main and sensitivity analysis were deemed significant.

#### Cladogram

We used the cladogram functionality of the Linear discriminant analysis Effect Size (LEfSe) method^41^ as implemented in Galaxy^42^ to illustrate differences in saliva microbiota composition between 6 and 24 months. In the cladogram, differences between the two time-points are illustrated at phyla, class, order, family, and genus levels.

#### Ethical approval

This study was approved by the Regional Ethical Review Board in Stockholm, Sweden (DNR 2011/192-31/2, with addendums), and all research was performed in accordance with all relevant guidelines and regulations. All women in the Born into Life cohort gave informed consent at enrolment during pregnancy, and both parents gave additional informed consent for the child.

## Results

The final study population comprised of 59 children. Maternal median age at birth was 31.9 years (Table 2). The majority of mothers had a university level education, were nulliparous, and had a normal BMI during the first trimester. None reported smoking during pregnancy. Of the 59 children, 22 were girls and 37 were boys. Overall, 12 children were delivered with a caesarean section. Three children were born preterm (<37 weeks) and two post-term (≥42 weeks). The median birth weight was 3560 grams.

**Table 2:** Baseline characteristics of the 59 mother-child pairs included in the study

Maternal characteristics	
Age at childbirth, years (IQR)	31.9 (29.7–34.3)
University level education (%)	
Yes	51 (86)
No	8 (14)
BMI in the first trimester, kg/m^2^ (%)	
Underweight (<18.5)	2 (3%)
Normal weight (18.5–24.9)	41 (69%)
Overweight (25.0–29.9)	13 (22%)
Obese (≥30.0)	3 (5%)
Smoking during pregnancy (%)	0 (0)
Parity (%)	
Nullipara	41 (69)
Multipara	18 (31)
**Birth characteristics**	
Sex (%)	
Female	22 (37)
Male	37 (63)
Mode of delivery (%)	
Vaginal	47 (80)
Caesarean	12 (20)
Birth weight, grams (IQR)	3560 (3240–3825)
Gestational age (%)	
Preterm (<37 weeks)	3 (5%)
Full term (37–41 weeks)	54 (92%)
Post term (≥42 weeks)	2 (3%)

IQR: Interquartile range.

BMI: Body Mass Index.

At follow-up visits (Table 3), a dispensed antibiotic prescription within 90 days before the visit was noted for 6%, 3%, and 16% of the children (at ages 6, 12 and 24 months, respectively). The cumulative incidence of ever having been prescribed antibiotic treatment reached 28% at age 24 months, and the two types of antibiotics most commonly prescribed were the β-lactam antibiotics phenoxylmethylpenicillin and amoxicillin (65% and 15% of all prescriptions, respectively). A furry pet was present in 2, 19 and 31% of the households responding to this question (at ages 6, 12 and 24 months, respectively).

**Table 3:** Study population characteristics at follow-up

	6 months	12 months	24 months
Number of children	48	38	32
Age, months (IQR)	6.6 (6.1–7.0)	12.3 (11.8–12.8)	24.5 (23.9–24.7)
Antibiotic treatment within 90 days (%)	3 (6)	1 (3)	5 (16)
Antibiotic treatment, ever-use (%)	3 (6)	2 (5)	9 (28)
Morning salivary cortisol, nmol/L (IQR)	24.0 (19.0–34.0)^a^	28.5 (17.4–37.4)^b^	23.3 (14.4–30.8)^c^
Furry pet present in household (%)	1 (2)^d^	3 (19)^e^	4 (31)^f^
Age when exclusive breastfeeding was discontinued (%)*			
0–2 months	4 (7)		
3–4 months	14 (24)		
5 + months	31 (53)		

IQR: Interquartile range.

^a^n = 42.

^b^n = 34.

^c^n = 28.

^d^n = 42.

^e^n = 16.

^f^n = 13.

*Column percentages do not add to total due to missing data.

### Age of child

**Figure 2:**
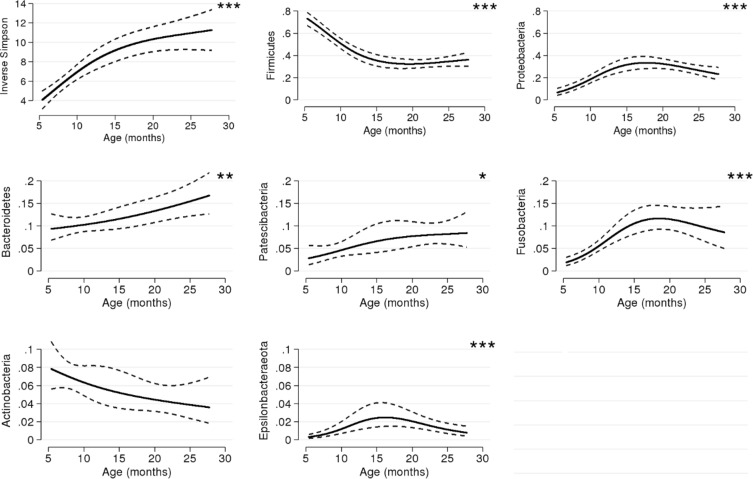
Model-based predictions of inverse Simpson and proportion of phyla plotted against age. Lines indicate mean values and 95% confidence intervals. The y-axis shows the relative abundance in proportion. *p < 0.05, **p < 0.01, ***p < 0.001.

**Figure 3:**
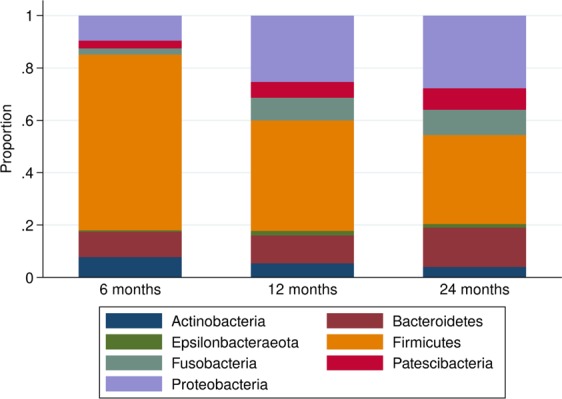
Stacked bar charts of phyla distribution at 6, 12, and 24 months, respectively.

**Figure 4:**
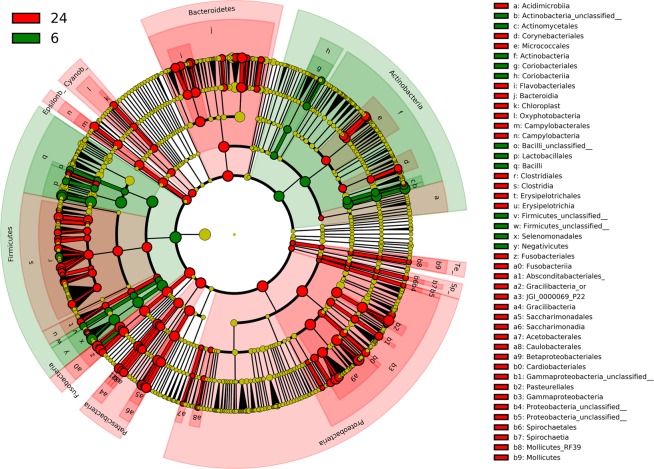
Cladogram showing differentially expressed bacteria at 6 and 24 months, respectively. Green colour indicates higher abundance at 6 months and red colour higher abundance at 24 months. Inner circle represents phyla and outer circles lower taxonomic levels.

Age of the child was positively associated with alpha diversity, with a nearly threefold increase noted from the first saliva sampling at 6 months to the sampling at 24 months (p < 0.001, Fig. 2). Increasing age was also associated with the overall distribution of phyla (p < 0.001), with the alterations in abundance of the phyla *Firmicutes*, *Proteobacteria*, *Epsilonbacteraoeta* (phyl. nov.) and *Fusobacteria* specifically identified in post-hoc analyses and also confirmed in semi-parametric sensitivity analyses as associated with age (all p < 0.001, Fig. 2 and Table S3). Overall, at 6 months, the phyla *Firmicutes* vastly dominated the oral microbiota, with a subsequent decrease noted both at 12 months and at 24 months (Fig. 3). The microbiota composition development between 6 and 24 months, across all investigated taxonomic levels, is depicted in the cladogram (Fig. 4).

**Figure 5:**
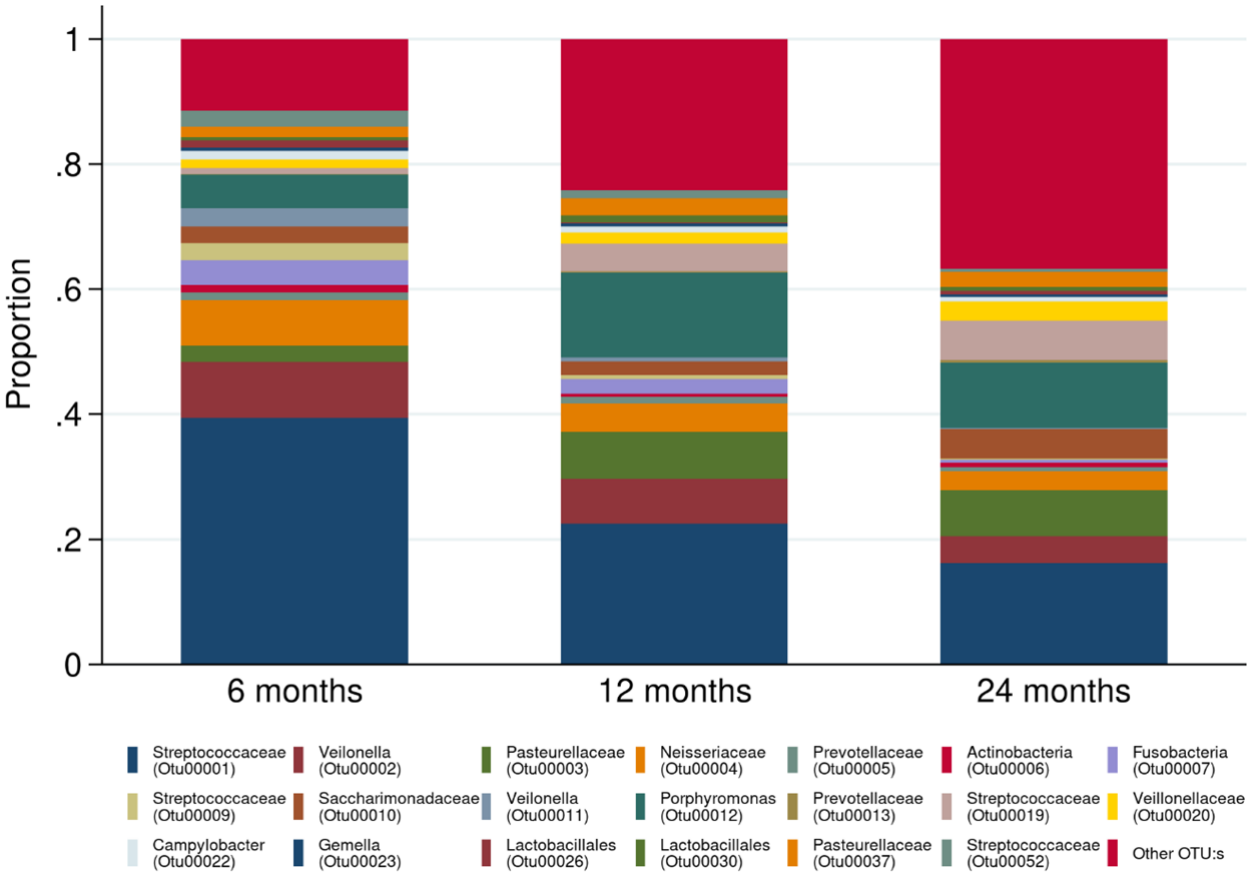
Stacked bar charts of the relative distribution of the 20 most abundant operational taxonomic units (OTUs) at 6, 12, and 24 months, respectively. Other OTUs constituted 11.5%, 24.3%, and 36.8% of the total amount of reads (at 6, 12 and 24 months respectively).

At the more highly resolved taxonomic levels, age was associated with 14 of the 20 most common OTUs explored (Fig. 5 and Fig. S2). The decreasing *Firmicutes* abundance noted at 12 and 24 months was here represented by steep reductions in OTUs belonging to the *Streptococcaceae* family (OTU0001, OTU0009, OTU00019, Table S2) and decreases of OTUs in the *Veillonella* genus or the *Veillonellaceae* family (OTU0002, OTU0011, and OTU0020, respectively). However, one initially rare OTU mapped to the Streptococcaceae family (OTU00052) increased with increasing age. Within the *Bacteroidetes* phyla, *Porphyromonas* (OTU00012) also increased with age, whereas two common OTUs within the *Proteobacteria* phyla belonging to the *Pasteurellaceae* (OTU00003) and *Neisseriaceae* (OTU00004) families, respectively, both exhibited inverted u-shaped associations with age. We furthermore noted an association between age and an OTU mapped as *Fusobacterium* genus (OTU0007).

**Figure 6:**
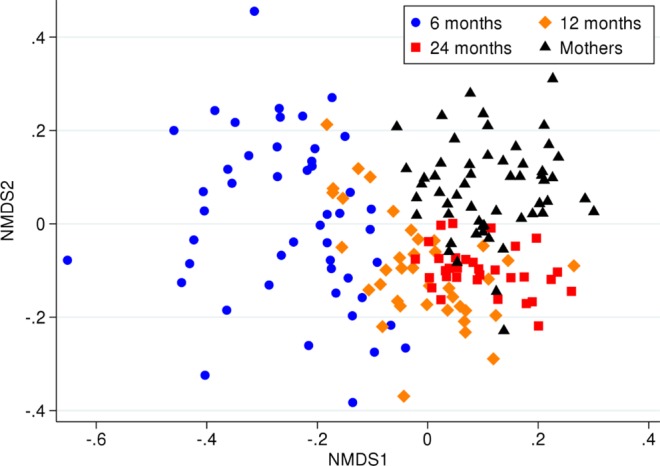
NMDS illustrating the children’s beta diversity across the three time points, as well as maternal beta diversity during pregnancy.

In addition, we also noted a decreased beta diversity (p < 0.001, Fig. 6).

### Maternal oral microbiota

The maternal oral microbiota composition during pregnancy was not associated with early childhood oral microbiota. However, when we compared the child cohort across all time points with the maternal cohort, we noted that that the children became more similar to an adult composition with increasing age (Fig. 6). Nevertheless, at 24 months, the microbiota of each child did not resemble their own mother’s microbiotaduring pregnancy more than any of the other samples of the maternal cohort (p = 0.17).

### Birth characteristics and breastfeeding

Gestational age and exclusive breastfeeding wereboth associated with overall phyla distribution (p < 0.01, Table S2). However, no specific phyla were identified in post-hoc analyses that could subsequently be confirmed in semi-parametric sensitivity analyses (Table S3). Gestational age and exclusive breastfeeding were also associated with beta diversity (p = 0.003 and p = 0.02, respectively).

**Figure 7:**
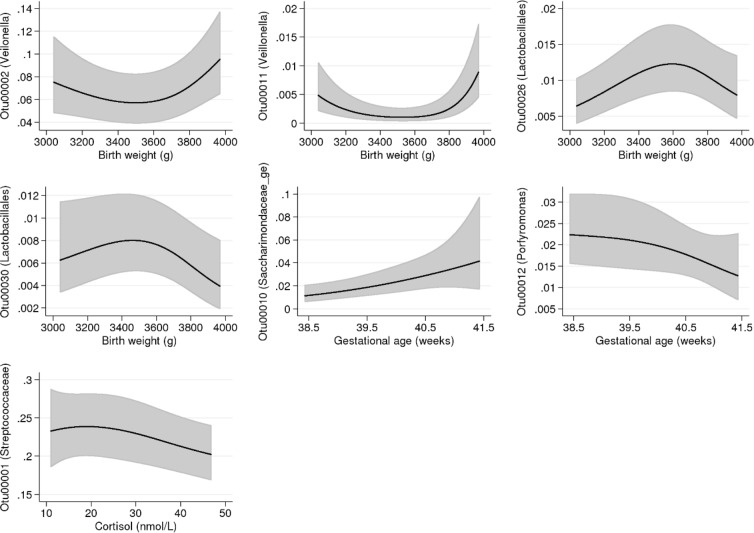
Relative abundance of specific operational taxonomic units (OTUs) associated with the continuous variables birth weight, gestational age and morning salivary cortisol. Lines indicate predicted mean values and 95% confidence intervals, adjusted for age and potential confounders (see Table 1). All continuous adjustment variables are fixed at their mean value and all categorical adjustment variables are fixed at their most common category. The x-axis ranges from the 10th to the 90th percentile of the variable. The y-axis shows the relative abundance in proportion.

Being born at an older gestational age was associated with an increase in the relative abundance of an OTU within the *Saccharimonadacea* genus (OTU00010, p < 0.001) but with a decrease of an OTU belonging to the *Porphyromonas* genus (OTU00012, p = 0.01, Fig. 7). Birth weight exhibited u-shaped associations with two OTUs within the *Veillonella* genus (OTU0002, p = 0.003 and OTU00011, p < 0.001). We also observed inverted u-shaped associations between birth weight and two OTUs belonging to the *Lactobacillales* order (OTU00026, p = 0.001 and OTU00030, p = 0.02, Fig. 7). We furthermore observed an interaction between birth weight and age for the presence of one of the *Veillonella* OTUs (OTU00011), where a birth weight below the 50^th^ percentile entailed a lower abundance noted foremost at 6 months of age (p < 0.001: Fig. S3). We found no associations between parity or mode of delivery and oral microbiota of the child.

### Morning salivary cortisol, antibiotic treatment, and pet exposure

Overall, we observed similar median morning salivary cortisol levels across all time points. Both morning salivary cortisol level and antibiotic treatment within the last 90 days of the saliva microbiota assessments were associated with proportion of phyla (p = 0.003 and p = 0.008, respectively), but no specific phyla were identified in post-hoc analyses that could also be confirmed in semi-parametric sensitivity analyses.

Morning salivary cortisol was inversely associated the relative abundance of the most common OTU mapped to the *Streptococcaceae* family (OTU0001, p = 0.02). This association was mainly driven be the group of children with cortisol values over 25 nmol/liter (Fig. 7).

Antibiotics treatment within the last 90 days was positively associated with two OTUs belonging to the *Pasteurellaceae* and *Neisseriaceae* families (OTU00003, RPR 1.84; p = 0.005 and OTU00004, RPR 1.84; p = 0.01, respectively) but negatively associated with an OTU within the *Prevotellaceae* family (OTU00013, RPR 0.07; p < 0.001). For the *Prevotellaceae* OTU we further noted an interaction between antibiotics prescribed within 90 days and age (OTU00013, p = 0.03: Fig. S3), with the lower abundance noted mainly at six months. We observed no association between ever-use of antibiotics and oral microbiota outcomes. Lastly, presence of a furry pet in the household was associated with a lower relative abundance of an OTU belonging to the *Pasteurellaceae* family (OTU00003, RPR 0.46; p = 0.01).

## Discussion

In this longitudinal population-based cohort study of 59 Swedish children, our main findings were twofold. First, we observed a radical shift in the salivary microbiota composition from 6 months to 24 months of age, including a nearly threefold increase in alpha diversity as well as profound changes on all investigated taxonomic levels. Secondly, we found a novel link between salivary cortisol and oral microbiota composition, encompassing both phyla distribution and lower relative abundance of the most common oral OTU belonging to the *Streptococcaceae* family. In addition, we also found evidence of associations between birth characteristics as well as other early life exposures with oral microbiota composition. We specifically noted a transient association between antibiotic treatment and the oral microbiome.

We observed a steady increase of oral microbiota alpha diversity associated with increasing age, indicating that both richness and species distribution evenness in individual saliva samples increased over time. This finding aligns with a recent Swedish longitudinal study of children with a family history of allergic disease that reported a positive association between age and oral microbiome alpha diversity^17^. At 6 months, we observed a vast dominance of *Firmicutes*, largely attributed to members of the Streptococci family. This is concordant with previous studies reporting a *Firmicutes* dominance at 4–8 weeks^14^, 3 months^15^, and 6 months of age^16^. The subsequent decrease of *Firmicutes*, accompanied by an increase in other phyla including *Proteobacteria*, also corresponds with the findings of two earlier studies which utilized pyrosequencing to examine saliva samples from 3-year old children^3,15^. We further found that the beta diversity of the children decreased with age and also that children’s samples became more similar to that of the maternal cohort over time, suggesting both that the initially larger inter-individual differences were attenuated over time, and that the children had reached a more adult-like composition at 24 months. This oral microbiota maturation process is likely influenced by several factors, including the emergence of deciduous teeth which creates new micro-environments and niches for oral bacterial colonization and microfilm adherence^43^ and changes in diet including the introduction of solid foods and an increase in variation of diet^44^. Additionally, an increased horizontal transmission may also occur as the child is increasingly exposed to a greater multitude of new people and environments, including day-care, during the first years of life. In Sweden, children start day-care at 12 months or later. On the OTU-level, we specifically noted increased relative abundances of OTUs within the *Porphyromonas* and the *Fusobacterium* genera with increasing age. The *Porphyromonas* genus include *Porphyromonas gingivalis*, implicated in periodontal disease but also previously detected in oral cavities of healthy children^45^, whilst the *Fusobacterium genus* include the bacteria *Fusobacterium nucleatum*, a species widely present in the oral cavity but also considered to be an opportunistic oral pathogen^46^. However, as our analysis pipe-line did not allow for species-level taxonomic resolution, we cannot discern the presence of these, or other potentially pathogenic, bacterial species within our samples.

We noted temporal similarities between our findings and previous reports on the development of the gut microbiota composition across the first years of life. As in the oral cavity, alpha diversity increases dramatically in the gut before the age of 24 months, with an adult-like composition reached at 2–3 years^47–49^. A longitudinal study of European and US children reported three distinct phases of gut microbiome progression, with an initial rapid development phase (3–14 months) characterized by major changes in the abundance of all five main gut phyla, a transitional phase (15–30 months) with distinct changes found only for two of the five main phyla, and a stable phase (>30 months) where little phyla redistribution occurred^50^. In correspondence, we observed the steepest changes in our two most abundant oral phyla *Firmicutes* and *Proteobacteria* in the first year of life, whilst less abrupt changes transpired in the children’s second year.

We could not detect any association between mode of delivery and oral microbiota. Caesarean sections has previously been noted as an important determinant for oral microbiota composition in newborns^12^. Our findings however align with the results from a longitudinal 16 S rRNA gene sequencing study of 81 infants, which did observe an association immediately after birth, but which could not detect any association at follow-up sampling at 6 weeks of age^11^. In contrast, other studies have reported that caesarean section may be associated with a lower number of oral bacterial taxa at 3 months^51^, and also with beta diversity at 6 months^17^, although the latter association was largely attenuated after adjustment for age, breastfeeding and later caries status. The discrepant results on the potential association between mode of delivery and oral microbiota likely reflect both that the influence of mode of delivery diminish over time and are therefore not detectable in our cohort where the first saliva sampling was performed at 6 months of age, as well as methodological dissimilarities between different study designs including adjustments for potential confounders.

We noted an association between duration of exclusive breastfeeding and a difference in overall phyla distribution, but could not identify any specific phyla or OTU associated with exclusive breastfeeding. Breastfeeding has previously been associated with abundance of *Actinobacteria* and Proteobacteria at 4–8 weeks^14^, and prevalence of *Lactobacillus* at 3 months^52^. An important limitation of our material is however that the upper pre-determined age-category for exclusive breastfeeding was 5+ months in the Born into Life questionnaire. Even children who were exclusively breast-fed for 5+ months may therefore have been introduced to solid foods and/or formula at the time of the first saliva sampling (at 6 months). Overall, the children in our cohort were also older and more likely to have deciduous teeth. The potential influence of breastfeeding on the oral microbiota has thereby likely been attenuated in our study. In addition, there are again also methodological differences between the previous studies and ours, as we have adjusted the statistical model for exclusive breastfeeding for several potential confounders identified through our DAG (Table 1), covariates that have not been included in the previous studies.

We observed that gestational age was associated with overall phyla distribution, and both gestational age and birth weight were associated with specific OTUs, including an inverted u-shaped associations between birth weight and two OTUs belonging to the *Lactobacillales* order. An American cohort study of 40 neonates recently reported that children born at a young gestational age with a low birth weight exhibited a greater abundance of the two genera *Stenotrophomonas* and *Enterobacter*, both within the *Gammaproteobacteria* class, as well as *Lactococcus*, a bacterial genus within the *Lactobacillales* order, as compared with term-born infants^9^. Although both studies note an influence of birth weight and gestational age on bacterial taxa within the *Lactobacillales* order, direct comparisons between the two studies may not be feasible as the American study only included very preterm infants sampled at the neonatal intensive care unit. In our study, being born at an older gestational age was also associated with an increase in the relative abundance of an OTU within the *Saccharimonadacea* genus (OTU00010), a genus belonging to the *Patescibacteria* phyla, a newly defined major microbial group within the updated topology of the bacterial domain^53^. No cultured isolates are available of the *Saccharimonadacea* genus as it has only been encountered through sequencing, and its function remains unclear. A recent study reported a higher abundance of *Saccharimonadacea* in non-caries afflicted adults than in adult with caries^54^. Further studies will however be needed to interpret our finding in association with gestational age.

To the best of our knowledge, no previous study has explored the association between naturally occurring variation in salivary cortisol and oral microbiota community composition. We observed that increased level of cortisol was associated with changes in phyla distribution, and a lower relative abundance of a common OTU belonging to the *Streptococcaceae* family. The association with the *Streptococcaceae* OTU was mainly seen in children with cortisol concentrations >25 nmol/L. Healthy Norwegian children have been reported to have a mean salivary cortisol of 28.7 nmol/L at 6 months, and 34.9 nmol/L at 24 months^55^. Cortisol has previously been found to alter *in vitro* growth of periodontal-related bacterial strains^56^. Our findings support that salivary cortisol is also associated with oral microbiota community composition *in vivo*.

Although we both applied a false discovery rate threshold, and filtered out results based on the more robust ordinal logistic regression in our analyses, we cannot exclude the possibility that random chance alone was responsible for our noted associations between salivary cortisol and oral microbiota. Further studies are needed to confirm our finding. We also adjusted all analyses of the associations between salivary cortisol and microbiota for the potential confounders available in the Born into Life study including maternal education level and birth characteristics, but we did not have access to information on other possible confounders including sleep patterns and dietary timing, as well as home and childcare environmental circumstances. Residual confounding may therefore still be present. Further, even though it may be of interest to investigate the association between cortisol circadian rhythm and saliva microbiota, we did not include measurements of evening salivary cortisol in our study in order minimize the risk of a matrix effect. Lastly, our study is cross-sectional in nature, and though we primarily hypothesize that cortisol may influence microbiota composition and development we cannot attempt to infer any causal direction. A reverse causation where oral microbiota may influence neuroendocrine activity and cortisol secretion remains possible.

If our associations are truly causal, we hypothesize that there could be different, potentially non-exclusive, pathways linking cortisol with oral bacterial taxa. The concept of a microbiome-gut-brain axis, with bidirectional communication between neuroendocrine activation including the hypothalamic-pituitary-adrenal axis and gut microbiota composition and function, is currently discussed within the gut microbiota research field^57–60^. Suggested mechanisms for the microbiome-gut-brain axis include a microbiota-driven pro-inflammatory activation of the hypothalamic-pituitary-adrenal axis, and an influence of cortisol on gut microbiota composition and gastrointestinal permeability. In parallel to this, we speculate that our results could indicate that the oral microbiota composition and development in early childhood may be influenced by systemic neuroendocrine activation. Another potential mechanism is that salivary cortisol could influence bacterial growth and biofilm formation in the oral cavity through local immunomodulation. Although longitudinal data is lacking, smaller cross-sectional studies have reported that salivary cortisol may be associated with the development of dental caries in childhood, and also with a larger number of cariogenic bacteria^19–21^, the latter including *Streptococcus mutans* and *Lactobacillus* species, bacteria involved in tooth decay and caries progression, respectively. Unfortunately, due to limitations in our analysis pipeline, we cannot discern if the cortisol-associated *Streptococcaceae* OTU in the present study represents *Streptococcus mutans*.

We further found that exposure to antibiotics within 90 days of saliva sampling was associated with proportion of phyla, as well as positively associated with OTUs belonging to the *Pasteurellaceae* and *Neisseriaceae* families and negatively associated with a OTU within the *Prevotellaceae* family. The most common indication for antibiotic treatment in young children in Sweden is a respiratory tract infection^61,62^ including acute otitis media, with national guidelines recommending treatment with a β-lactam antibiotics for 5–10 days. Although we do not have access to treatment duration for the children in the Born into Life cohort, phenoxylmethylpenicillin and amoxicillin represented the most commonly prescribed antibiotics mirroring the national guidelines. In interpreting our findings, it is also noteworthy that antibiotics are most often prescribed as oral suspensions to young children, with potential local effects in the oral cavity.

The *Pasteurellaceae* and *Neisseriaceae* families contain known pathogens including *Pasteurella multocida* and *Neisseria meningitidis*. However, although we cannot determine if these species are included in the OTUs we have detected, these pathogens are sensitive to phenoxylmethylpenicillin and amoxicillin, and should not have survived the antibiotics treatment. A Swiss cohort study using pyrosequencing found that amoxicillin prescribed for otitis media in children was associated with a reduced species richness and diversity in the oral cavity, and with a major shift in relative abundance of several taxa at the end of the antibiotic treatment^63^. However, three weeks after the amoxicillin treatment ended, the saliva microbiota had largely recovered to the composition present before treatment commenced^63^. Corresponding results were reported from a randomized controlled trial on healthy adults^64^, which found that antibiotic treatment had extensive and long-term effects on gut microbiota, whereas alternations of oral microbiota after exposure to antibiotics were of lower magnitude and transient. A swift recovery of oral microbiota after antibiotic treatment cessation may explain why we could not distinguish any association between ever-use of antibiotics and oral microbiota in our cohort.

Lastly, we also noted that presence of a furry pet in the household was associated with a lower relative abundance of an OTU within the *Pasteurellaceae* family, a bacterial family containing strains that are potential zoonotic agents and are usually present in cats and dogs. The direction of the association was surprising and further studies are needed to confirm the finding.

### Strengths and limitations

A strength of this study is the longitudinal design, which allowed for repeated assessments of oral microbiota and salivary cortisol, as well as for time-updated information on early childhood exposures. Linkages to national health registers furthermore enabled access to objectively recorded maternal and birth characteristics, and also to detailed information on antibiotic prescription without any risk of recall bias. The identification of potential confounders for each exposure before the analysis phase of the study further reduced the overall risk of bias.

In addition to the limitations discussion in association with our findings on salivary cortisol, a few more potential weaknesses of our study however also deserve mentioning. Firstly, our platform for 16 s rRNA amplicon sequencing did not allow for a species-level taxonomic resolution in OTUs. Interpreting potential pathogenic effects of our associations is therefore challenging. Secondly, the time-points for questionnaires and biomarker sampling were pre-determined for the Born into Life cohort, and unfortunately no saliva sampling was performed in the neonatal period. Further, our sample size of 59 mother-child pairs had limited power to detect associations for rarer exposures such as caesarean section, antibiotic treatment and furry pet in our cohort, and also to identify changes in specific phyla contributing to an overall change in phyla distribution. Also, fewer children participated in the examinations at 12 and 24 months than at 6 months which may have further reduced the possibility to detect associations present only at older age. Finally, the maternal cohort represents a well-educated population who had originally volunteered to participate in LifeGene and then also accepted to partake in Born into Life whilst pregnant, and the child cohort consists of mainly term-born healthy children. This limited our possibility to observe for example associations between unfavourable maternal health behaviours and the child’s oral microbiota, and may also restrict the generalizability of our findings. However, expanding the knowledge base of the oral microbiota development trajectory in a healthy context may enable future identification of adverse oral microbiota markers in children born in less advantageous environments.

In conclusion, the findings of this longitudinal study include a novel association between salivary cortisol and oral microbiota and a transient effect of antibiotics on the oral microbiome in early childhood. It also highlights the radical shifts in the oral microbiota composition from 6 months to 24 months of age. Future studies utilizing metagenome shotgun sequencing may enable an increased accuracy and a more detailed information on bacterial function.

## Supplementary information

Supplementary material

## Data Availability

Restrictions apply to the availability of individual level health data, which were used under license and ethical approval for the current study, and are not publicly available. Individual level data are however available from the authors upon reasonable request and with permission of the Swedish Ethical Review Authority. Bacterial sequencing data has been submitted to the Sequence Read Archive (SRA) open repository under reference number PRJNA575550.
