# Supplementary material for: Oral Microbiota Development in Early Childhood
Source: Sci Rep. 2019 Dec 13;9:19025. doi: 10.1038/s41598-019-54702-0 (PMC6911045; doi:10.1038/s41598-019-54702-0)
Supplement: Supplementary file 1 — Supplementary material [file 41598_2019_54702_MOESM1_ESM.pdf]

ORAL MICROBIOTA DEVELOPMENT IN EARLY CHILDHOOD

Beatrice Kennedy[1*], Sari Peura[1, 2], Ulf Hammar[1], Silvia Vicenzi[1,3], Anna Hedman[4], Catarina Almqvist[4, 5], Ellika Andolf[6], Göran Pershagen[7], Johan Dicksved[8], Stefan Bertilsson[9], Tove Fall[1].

[1] Department of Medical Sciences, Molecular Epidemiology and Science for Life Laboratory, Uppsala University, Uppsala, Sweden.

[2] Department of Forest Mycology and Plant Pathology, Science for Life Laboratory, Swedish University of Agricultural Sciences, Uppsala, Sweden.

[3] School of Medicine, University of Tasmania, Hobart, Australia.

[4] Department of Medical Epidemiology and Biostatistics, Karolinska Institutet, Stockholm, Sweden.

[5] Unit of Pediatric Allergy and Pulmonology at Astrid Lindgren Children's Hospital, Karolinska University Hospital, Stockholm, Sweden.

[6] Department of Clinical Sciences, Danderyd Hospital, Stockholm, Sweden.

[7] Institute of Environmental Medicine, Karolinska Institutet, Stockholm, Sweden.

[8] Department of Animal Nutrition and Management, Swedish University of Agricultural Sciences, Uppsala, Sweden.

[9] Department of Ecology and Genetics, Limnology, Uppsala University, Uppsala, Sweden.

* Corresponding author: beatrice.kennedy@medsci.uu.se

SUPPLEMENTARY INFORMATION

Saliva sample instructions

Parents were instructed to take the first saliva sample from their child the evening before their planned visit to the test centre, just before bedtime but not within 30 minutes of eating or brushing their teeth. The second saliva sample was to be collected the same morning as their visit, within 15 minutes after waking up and before the first meal of the day. Parents were given the sampling kit by the test centre and were instructed to let the child suck on a cotton swab for one minute to saturate it with saliva and then to store it in a plastic tube, marked with the date and time, at standard refrigerator temperature. Mothers followed similar instructions at sampling of their saliva during pregnancy.

PCR protocol

The v3 and v4 regions of 16S rRNA gene were amplified using a two-step PCR protocol. In the first PCR, 16S rRNA target region was amplified using primers 341F and 805R [24]. The protocol included initial denaturation at 98 ˚C for 30 s, followed by 20 cycles of 98 ˚C for 10 s, 62 ˚C for 30 s and 72 ˚C for 30 s. Final elongation was 2 min at 72 ˚C. After purification with AMPure beads (Agencourt) following manufacture protocol, a second PCR was performed to add sample-specific DNA barcodes. This PCR included initial denaturation at 98 ˚C for 30 s, followed by 15 cycles of 98 ˚C for 10 s, 66 ˚C for 30 s and 72 ˚C for 30 s and final elongation was 2 min at 72 ˚C.

SUPPLEMENTARY FIGURES

Figure S1.

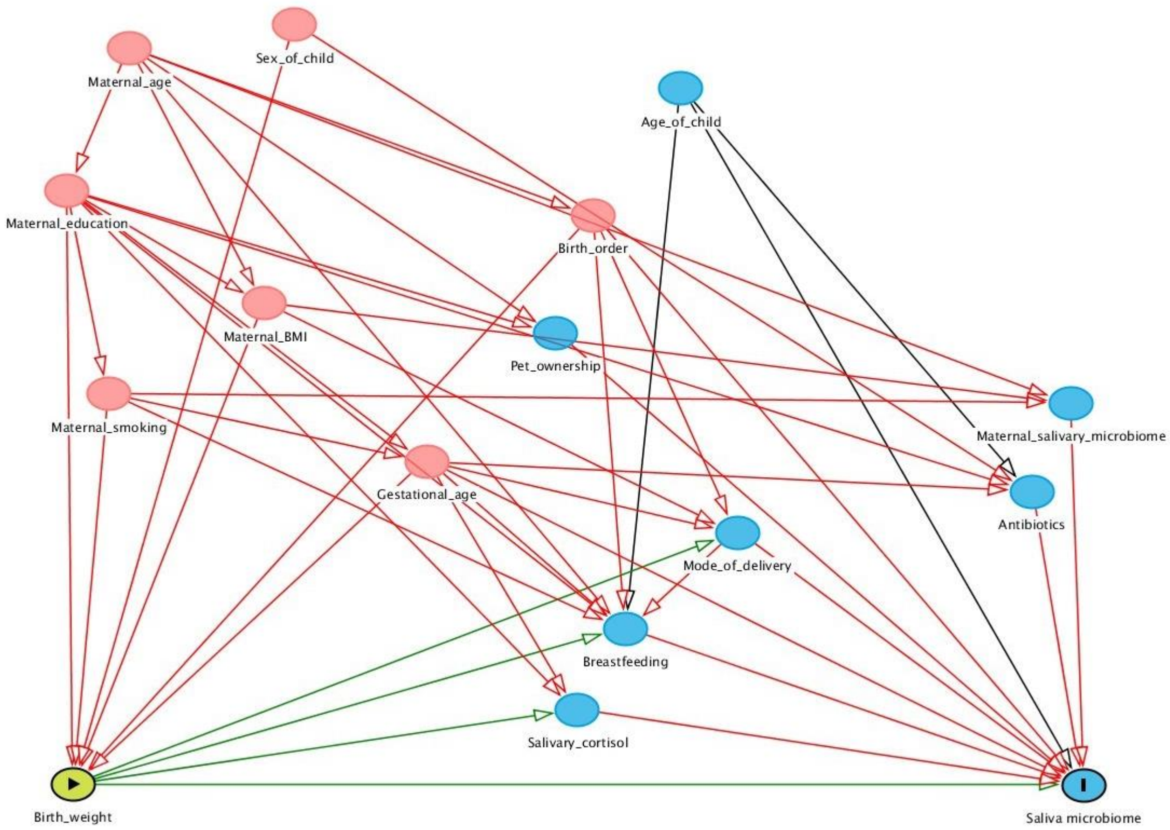

Directed acyclic graph of theoretical framework.

Figure S2.

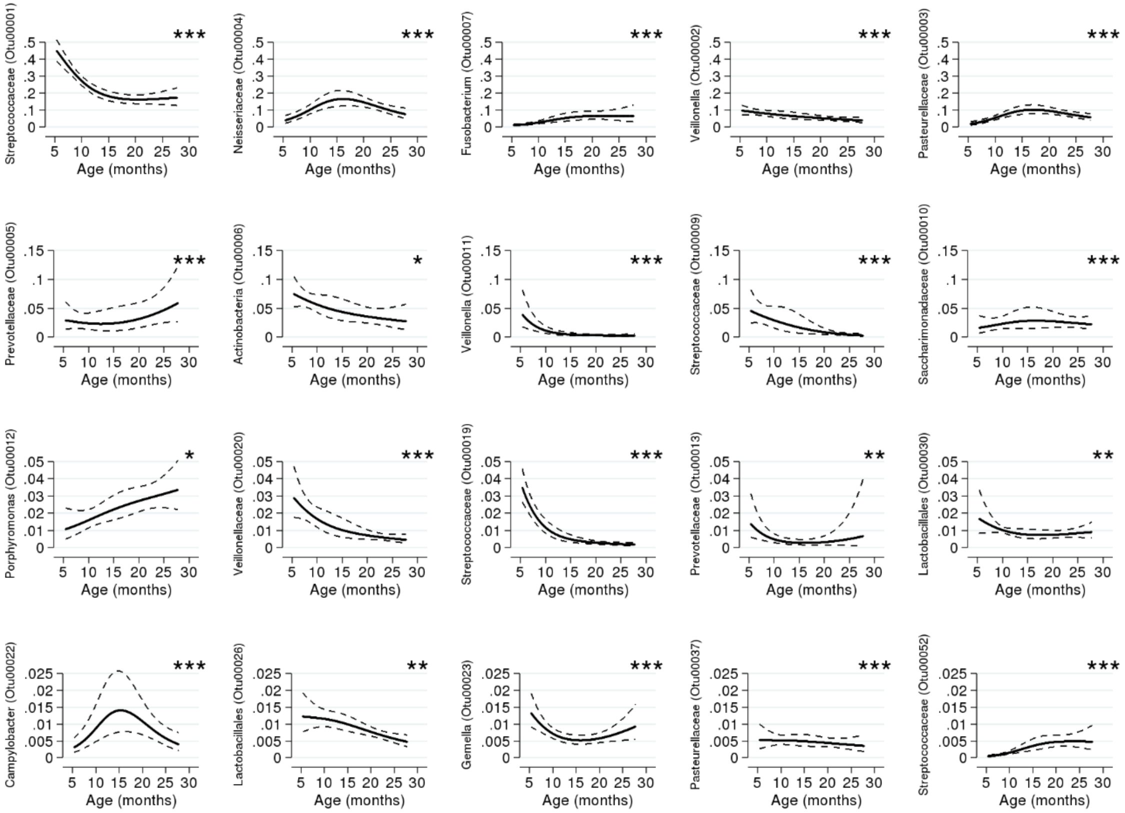

Relative abundance of the 20 most common OTUs plotted against age. Lines indicate mean values and 95% confidence intervals. The y-axis shows the relative abundance in proportion. * p<0.05, ** p<0.01, *** p<0.001.

Figure S3.

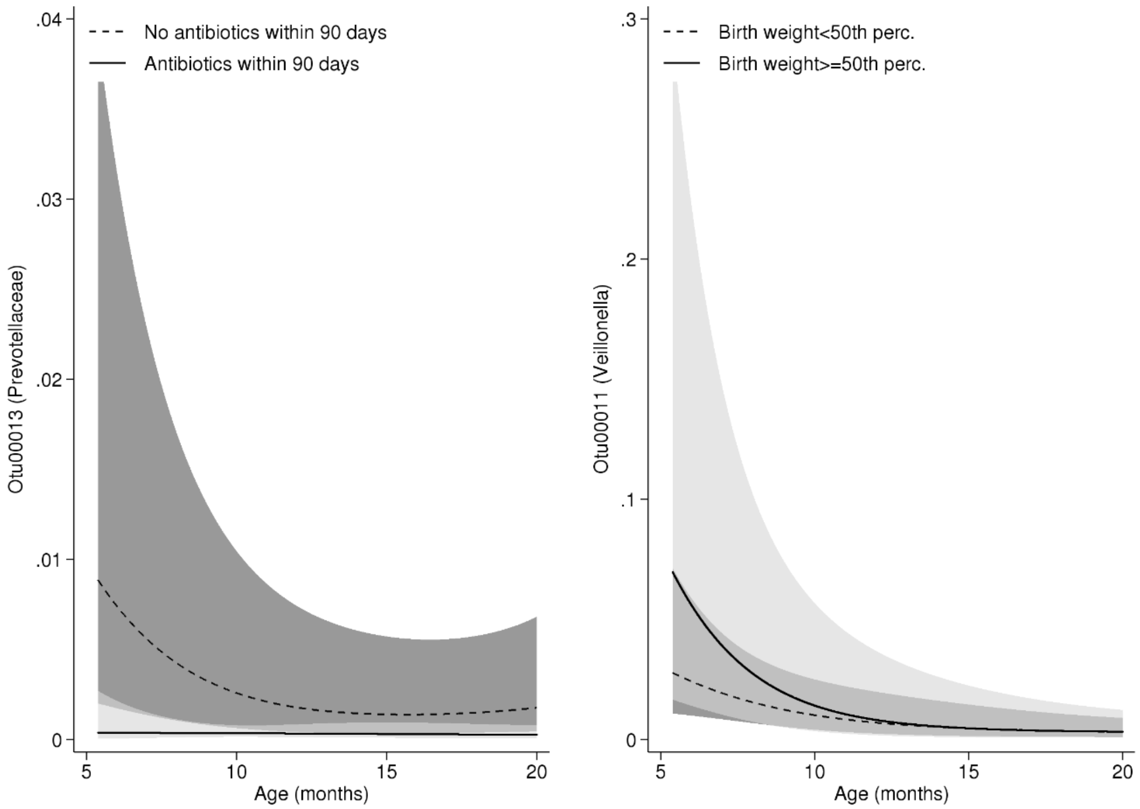

The interactions between antibiotics within 90 days, the child's age, and an OTU within the Prevotellaceae family, and birth weight, the child's age, and an OTU within the Veillonella genus, respectively. The y-axis shows the relative abundance in proportion.

SUPPLEMENTARY TABLES

Supplementary table 1. The 20 most common operational taxonomic units (OTUs)

OTU number	Highest resolved taxonomic level	Genus	Family	Order	Class	Phylum
Otu00001	Family	-	Streptococcaceae	Lactobacillales	Bacilli	Firmicutes
Otu00002	Genus	Veillonella	Veillonellaceae	Selenomonadales	Negativicutes	Firmicutes
Otu00003	Family		Pasteurellaceae	Pasteurellales	Gammaproteobacteria	Proteobacteria
Otu00004	Family	-	Neisseriaceae	Betaproteobacteriales	Gammaproteobacteria	Proteobacteria
Otu00005	Genus	-	Prevotellaceae	Bacteroidales	Bacteroidia	Bacteroidetes
Otu00006	Class	-	-	-	Actinobacteria	Actinobacteria
Otu00007	Genus	Fusobacterium	Fusobacteriaceae	Fusobacteriales	Fusobacteriia	Fusobacteria
Otu00009	Family	-	Streptococcaceae	Lactobacillales	Bacilli	Firmicutes
Otu00010	Genus	Saccharimonadaceae	Saccharimonadaceae	Saccharimonadales	Saccharimonadia	Patescibacteria
Otu00011	Genus	Veillonella	Veillonellaceae	Selenomonadales	Negativicutes	Firmicutes
Otu00012	Genus	Porphyromonas	Porphyromonadaceae	Bacteroidales	Bacteroidia	Bacteroidetes
Otu00013	Family		Prevotellaceae	Bacteroidales	Bacteroidia	Bacteroidetes
Otu00019	Family	-	Streptococcaceae	Lactobacillales	Bacilli	Firmicutes
Otu00020	Family		Veillonellaceae	Selenomonadales	Negativicutes	Firmicutes
Otu00022	Genus	Campylobacter	Campylobacteraceae	Campylobacterales	Campylobacteria	Epsilonbacteraeota
Otu00023	Genus	Gemella	Family_XI	Bacillales	Bacilli	Firmicutes
Otu00026	Order	-	-	Lactobacillales	Bacilli	Firmicutes
Otu00030	Order	-	-	Lactobacillales	Bacilli	Firmicutes
Otu00037	Family	-	Pasteurellaceae	Pasteurellales	Gammaproteobacteria	Proteobacteria
Otu00052	Family	-	Streptococcaceae	Lactobacillales	Bacilli	Firmicutes

Supplementary table 2. Results from regression analyses.

Exposure	Outcome	p-value main analysis	Direction	N	p-value sensitivity analysis	Significant †
**Age (months)**	Alpha diversity (inverse Simpson)	1x10-13		59	3x10-12	1
	Beta diversity (NMDS)	6x10-61		59	6x10-36	1
	OTU00001	3x10-16		59	2x10-10	1
	OTU00002	2x10-4		59	5x10-3	1
	OTU00003	2x10-6		59	4x10-10	1
	OTU00004	2x10-4		59	3x10-7	1
	OTU00005	0.23		59	0.02	0
	OTU00006	0.02		59	5x10-3	1
	OTU00007	2x10-7		59	3x10-10	1
	OTU00009	3x10-10		59	1x10-3	1
	OTU00010	0.66		59	1x10-4	0
	OTU00011	2x10-7		59	6x10-4	1
	OTU00012	0.01		59	3x10-8	1
	OTU00013	3x10-3		59	0.97	0
	OTU00019	1x10-29		59	1x10-15	1
	OTU00020	5x10-9		59	2x10-3	1
	OTU00022	6x10-5		59	1x10-4	1
	OTU00023	8x10-4		59	0.40	0
	OTU00026	1x10-3		59	9x10-3	1
	OTU00030	0.17		59	0.37	0
	OTU00037	0.56		59	0.04	0
	OTU00052	8x10-12		59	6x10-10	1
	Proportion of phyla	3x10-32		59	6x10-21	1
**Antibiotics (ever-use)**	Alpha diversity (inverse Simpson)	0.13	-	59	0.48	0
	Beta diversity (NMDS)	0.25		59	0.06	0
	OTU00001	0.99	+	59	0.74	0

	OTU00002	0.94	-	59	0.34	0
	OTU00003	0.11	+	59	0.14	0
	OTU00004	0.03	+	59	0.10	0
	OTU00005	0.44	-	59	0.51	0
	OTU00006	0.77	-	59	0.98	0
	OTU00007	0.42	-	59	0.36	0
	OTU00009	0.01	-	59	0.10	0
	OTU00010	0.79	-	59	0.22	0
	OTU00011	0.32	+	59	0.90	0
	OTU00012	0.51	+	59	0.88	0
	OTU00013	9x10-4	-	59	0.07	0
	OTU00019	0.97	-	59	0.94	0
	OTU00020	0.02	-	59	0.13	0
	OTU00022	0.36	-	59	0.27	0
	OTU00023	0.65	-	59	0.96	0
	OTU00026	0.75	-	59	0.92	0
	OTU00030	6x10-3	-	59	0.12	0
	OTU00037	0.47	-	59	0.73	0
	OTU00052	0.63	+	59	0.04	0
	Proportion of phyla	0.29		59	0.54	0
**Antibiotics (within 90 days)**	Alpha diversity (inverse Simpson)	0.66	-	59	0.91	0
	Beta diversity (NMDS)	0.05		59	0.01	0
	OTU00001	0.97	-	59	0.60	0
	OTU00002	0.33	+	59	0.57	0
	OTU00003	5x10-3	+	59	0.03	1
	OTU00004	0.01	+	59	0.05	1
	OTU00005	0.02	-	59	0.53	0
	OTU00006	0.67	+	59	0.62	0
	OTU00007	0.84	+	59	0.90	0
	OTU00009	0.03	-	59	0.75	0

	OTU00010	0.87	+	59	0.62	0
	OTU00011	0.14	+	59	0.28	0
	OTU00012	0.45	+	59	0.92	0
	OTU00013	6x10-5	-	59	0.02	1
	OTU00019	0.79	+	59	0.16	0
	OTU00020	0.04	-	59	0.13	0
	OTU00022	0.82	-	59	0.57	0
	OTU00023	0.97	-	59	0.60	0
	OTU00026	0.78	+	59	0.37	0
	OTU00030	4x10-3	-	59	0.16	0
	OTU00037	0.79	-	59	0.83	0
	OTU00052	0.22	+	59	0.18	0
	Proportion of phyla	8x10-3		59	0.04	1
**Birth weight (gram)**	Alpha diversity (inverse Simpson)	0.17		59	0.35	0
	Beta diversity (NMDS)	0.14		59	0.35	0
	OTU00001	0.28		59	0.38	0
	OTU00002	3x10-3		59	0.05	1
	OTU00003	0.93		59	0.85	0
	OTU00004	0.37		59	0.68	0
	OTU00005	0.04		59	0.11	0
	OTU00006	0.52		59	0.58	0
	OTU00007	0.80		59	0.37	0
	OTU00009	0.71		59	0.27	0
	OTU00010	0.05		59	0.33	0
	OTU00011	7x10-13		59	1x10-4	1
	OTU00012	0.20		59	0.10	0
	OTU00013	0.07		59	0.03	0
	OTU00019	0.51		59	0.36	0
	OTU00020	0.81		59	0.33	0
	OTU00022	0.17		59	0.24	0

	OTU00023	9x10-3		59	0.31	0
	OTU00026	1x10-3		59	0.05	1
	OTU00030	0.02		59	4x10-3	1
	OTU00037	0.36		59	0.25	0
	OTU00052	4x10-9		59	0.07	0
	Proportion of phyla	2x10-5		59	0.09	0
**Exclusive breastfeeding**	Alpha diversity (inverse Simpson)	0.51	-	49	0.09	0
	Beta diversity (NMDS)	0.02		49	4x10-3	1
	OTU00001	0.36	+	49	0.28	0
	OTU00002	0.95	-	49	0.44	0
	OTU00003	0.68	-	49	0.60	0
	OTU00004	0.03	+	49	0.10	0
	OTU00005	0.07	-	49	0.14	0
	OTU00006	0.86	-	49	0.90	0
	OTU00007	0.98	-	49	0.21	0
	OTU00009	0.54	+	49	0.57	0
	OTU00010	8x10-3	-	49	0.52	0
	OTU00011	0.37	+	49	0.29	0
	OTU00012	0.82	+	49	0.54	0
	OTU00013	0.02	+	49	0.43	0
	OTU00019	0.53	+	49	0.23	0
	OTU00020	0.04	-	49	0.07	0
	OTU00022	0.94	-	49	0.49	0
	OTU00023	0.95	+	49	0.17	0
	OTU00026	3x10-3	+	49	0.11	0
	OTU00030	0.34	+	49	0.62	0
	OTU00037	4x10-3	+	49	0.16	0
	OTU00052	0.19	+	49	0.77	0
	Proportion of phyla	5x10-3		49	0.02	1

**Furry pet**	Alpha diversity (inverse Simpson)	0.15	+	49	0.44	0
	Beta diversity (NMDS)	0.54		49	0.95	0
	OTU00001	0.85	-	49	0.42	0
	OTU00002	0.57	-	49	0.70	0
	OTU00003	0.01	-	49	0.04	1
	OTU00004	0.98	+	49	0.91	0
	OTU00005	0.77	-	49	0.56	0
	OTU00006	0.03	-	49	0.03	0
	OTU00007	0.34	+	49	0.74	0
	OTU00009	0.06	+	49	0.73	0
	OTU00010	0.73	-	49	0.68	0
	OTU00011	0.34	+	49	0.56	0
	OTU00012	0.04	+	49	0.42	0
	OTU00013	0.07	+	49	0.58	0
	OTU00019	0.09	+	49	0.87	0
	OTU00020	0.10	-	49	0.64	0
	OTU00022	0.61	-	49	0.82	0
	OTU00023	0.38	-	49	0.68	0
	OTU00026	0.21	-	49	0.20	0
	OTU00030	0.58	-	49	0.21	0
	OTU00037	0.49	-	49	0.31	0
	OTU00052	0.79	-	49	0.51	0
	Proportion of phyla	2x10-5		49	0.27	0
**Gestational age (weeks)**	Alpha diversity (inverse Simpson)	0.23		59	0.28	0
	Beta diversity (NMDS)	3x10-3		59	6x10-7	1
	OTU00001	0.07		59	0.20	0
	OTU00002	0.25		59	0.27	0
	OTU00003	0.07		59	4x10-4	0
	OTU00004	0.57		59	0.42	0

	OTU00005	0.04	59	0.02	0
	OTU00006	0.27	59	0.80	0
	OTU00007	0.06	59	0.45	0
	OTU00009	0.19	59	0.97	0
	OTU00010	5x10-4	59	5x10-5	1
	OTU00011	0.80	59	0.55	0
	OTU00012	0.01	59	0.04	1
	OTU00013	0.52	59	0.04	0
	OTU00019	0.41	59	0.01	0
	OTU00020	0.64	59	0.95	0
	OTU00022	4x10-5	59	0.55	0
	OTU00023	0.28	59	0.31	0
	OTU00026	0.46	59	0.72	0
	OTU00030	0.66	59	0.02	0
	OTU00037	0.52	59	0.23	0
	OTU00052	0.06	59	0.65	0
	Proportion of phyla	2x10-21	59	2x10-14	1
**Maternal alpha diversity (inverse Simpson)**	Alpha diversity (inverse Simpson)	0.66	55	0.70	0
**Maternal beta diversity (NMDS1)**	Beta diversity (NMDS)	0.07	55	0.02	0
**Maternal beta diversity (NMDS2)**	Beta diversity (NMDS)	0.09	55	0.26	0
**Maternal OTU00001**	OTU00001	0.55	55	0.69	0
**Maternal OTU00002**	OTU00002	0.73	55	0.68	0
**Maternal OTU00003**	OTU00003	0.21	55	0.23	0
**Maternal OTU00004**	OTU00004	0.12	55	0.63	0
**Maternal OTU00005**	OTU00005	0.52	55	0.24	0
**Maternal OTU00006**	OTU00006	0.36	55	0.55	0
**Maternal OTU00007**	OTU00007	0.74	55	0.58	0
**Maternal OTU00009**	OTU00009	6x10-7	55	0.71	0
**Maternal OTU00010**	OTU00010	0.55	55	0.45	0

**Maternal OTU00011**	OTU00011	0.02		55	0.78	0
**Maternal OTU00012**	OTU00012	0.12		55	0.47	0
**Maternal OTU00013**	OTU00013	0.03		55	0.06	0
**Maternal OTU00019**	OTU00019	0.24		55	0.04	0
**Maternal OTU00020**	OTU00020	0.17		55	0.57	0
**Maternal OTU00022**	OTU00022	0.14		55	0.01	0
**Maternal OTU00023**	OTU00023	0.32		55	0.09	0
**Maternal OTU00026**	OTU00026	0.30		55	0.06	0
**Maternal OTU00030**	OTU00030	0.24		55	0.05	0
**Maternal OTU00037**	OTU00037	0.85		55	0.49	0
**Maternal OTU00052**	OTU00052	0.13		55	0.71	0
**Maternal proportion of phyla**	Proportion of phyla	0.12		55		0
**Mode of delivery**	Alpha diversity (inverse Simpson)	0.63	+	59	0.32	0
	Beta diversity (NMDS)	0.59		59	0.34	0
	OTU00001	0.90	-	59	0.66	0
	OTU00002	1.00	-	59	1.00	0
	OTU00003	0.77	+	59	0.52	0
	OTU00004	0.23	+	59	0.18	0
	OTU00005	0.05	+	59	0.02	0
	OTU00006	0.61	-	59	0.87	0
	OTU00007	0.10	+	59	0.42	0
	OTU00009	0.36	-	59	0.04	0
	OTU00010	0.52	-	59	0.08	0
	OTU00011	0.74	-	59	0.85	0
	OTU00012	0.42	+	59	0.41	0
	OTU00013	0.53	-	59	0.18	0
	OTU00019	0.69	+	59	0.51	0
	OTU00020	0.08	-	59	0.55	0
	OTU00022	0.29	+	59	0.44	0
	OTU00023	0.30	+	59	0.22	0

	OTU00026	0.49	-	59	0.65	0
	OTU00030	0.23	-	59	0.79	0
	OTU00037	0.08	-	59	0.78	0
	OTU00052	0.73	-	59	0.38	0
	Proportion of phyla	0.59		59	0.94	0
**Morning salivary cortisol**	Alpha diversity (inverse Simpson)	0.93		57	0.26	0
	Beta diversity (NMDS)	0.03		57	0.03	0
	OTU00001	0.02		57	0.01	1
	OTU00002	0.05		57	0.13	0
	OTU00003	0.37		57	0.71	0
	OTU00004	0.56		57	0.86	0
	OTU00005	4x10-3		57	0.08	0
	OTU00006	0.24		57	0.41	0
	OTU00007	0.11		57	0.99	0
	OTU00009	0.90		57	0.61	0
	OTU00010	0.42		57	0.28	0
	OTU00011	0.53		57	0.03	0
	OTU00012	0.11		57	0.12	0
	OTU00013	0.50		57	0.12	0
	OTU00019	0.54		57	0.71	0
	OTU00020	0.13		57	0.39	0
	OTU00022	0.41		57	0.80	0
	OTU00023	6x10-3		57	0.10	0
	OTU00026	0.50		57	0.72	0
	OTU00030	0.10		57	0.04	0
	OTU00037	0.56		57	0.46	0
	OTU00052	0.65		57	0.94	0
	Proportion of phyla	3x10-3		57	1x10-4	1
**Parity**	Alpha diversity (inverse Simpson)	0.66	+	59	0.61	0

	Beta diversity (NMDS)	0.98		59	0.93	0
	OTU00001	0.45	+	59	0.69	0
	OTU00002	0.79	-	59	0.99	0
	OTU00003	0.06	-	59	0.48	0
	OTU00004	0.14	+	59	0.25	0
	OTU00005	0.86	+	59	0.55	0
	OTU00006	0.74	+	59	0.32	0
	OTU00007	0.85	+	59	0.45	0
	OTU00009	0.59	-	59	0.83	0
	OTU00010	0.03	+	59	0.29	0
	OTU00011	0.08	-	59	0.95	0
	OTU00012	0.07	+	59	0.31	0
	OTU00013	0.20	-	59	0.69	0
	OTU00019	0.53	+	59	0.84	0
	OTU00020	0.65	-	59	0.24	0
	OTU00022	0.07	+	59	0.41	0
	OTU00023	0.86	+	59	0.20	0
	OTU00026	0.35	+	59	0.28	0
	OTU00030	0.33	-	59	0.99	0
	OTU00037	0.18	-	59	0.91	0
	OTU00052	0.58	-	59	0.28	0
	Proportion of phyla	0.92		59	0.99	0

† Significant result after FDR and sensitivity analysis. 1=yes. 2=no

Supplementary table 3. Post hoc tests for phyla distribution.

Exposure	Phylum	p-value main analysis	p-value sensitivity analysis
**Age**	Actinobacteria	0.35	0.04
	Bacteria_unclassifie	0.66	0.21
	Bacteroidetes	0.52	0.02
	**Epsilonbacteraeota**	**1x10-8**	**5x10-7**
	**Firmicutes**	**2x10-12**	**7x10-13**
	**Fusobacteria**	**3x10-7**	**6x10-13**
	Patescibacteria	0.15	2x10-5
	**Proteobacteria**	**7x10-9**	**2x10-14**
**Antibiotics (within 90 days)**	Actinobacteria	0.68	0.81
	Bacteria_unclassifie	0.58	0.40
	Bacteroidetes	0.18	0.15
	Epsilonbacteraeota	0.37	0.49
	Firmicutes	0.77	0.75
	Fusobacteria	0.91	0.84
	Patescibacteria	0.22	0.10
	Proteobacteria	0.05	0.18
**Birth weight**	Actinobacteria	0.23	0.35
	Bacteria_unclassifie	0.07	0.09
	Bacteroidetes	0.29	0.08
	Epsilonbacteraeota	0.26	0.20
	Firmicutes	0.76	0.31
	Fusobacteria	0.66	0.16
	Patescibacteria	0.61	0.44
	Proteobacteria	0.18	0.46
**Exclusive breastfeeding**	Actinobacteria	0.91	0.93
	Bacteria_unclassifie	0.34	0.37
	Bacteroidetes	0.43	0.66
	Epsilonbacteraeota	0.32	0.61
	Firmicutes	0.71	0.94

	Fusobacteria	0.28	0.11
	Patescibacteria	2x10-3	0.08
	Proteobacteria	0.04	0.23
**Morning salivary cortisol**	Actinobacteria	0.16	0.21
	Bacteria_unclassifie	0.06	2x10-3
	Bacteroidetes	0.09	0.15
	Epsilonbacteraeota	0.83	0.59
	Firmicutes	0.88	0.96
	Fusobacteria	0.11	0.62
	Patescibacteria	0.72	0.25
	Proteobacteria	0.30	0.34
**Gestational age**	Actinobacteria	0.19	0.81
	Bacteria_unclassifie	0.20	0.61
	Bacteroidetes	0.22	0.44
	Epsilonbacteraeota	8x10-4	0.40
	Firmicutes	0.49	0.22
	Fusobacteria	0.06	0.13
	Patescibacteria	0.05	0.11
	Proteobacteria	0.23	0.29

Supplementary table 4. Sensitivity regression analysis of beta diversity using PERMANOVA

Microbiota outcome	Exposure	p-value	N	Significance using PERMANOVA†	Significance in main analysis using NMDS*
**Beta diversity in child (PERMANOVA)**	Age	5x10-4	59	1	1
	Antibiotics (ever-use)	0.18	59	0	0
	Antibiotics (within 90 days)	0.24	59	0	0
	Birth weight	0.10	59	0	0
	Exclusive breastfeeding	0.02	49	1	1
	Furry pet	0.55	49	0	0
	Gestational age	0.26	59	0	1
	Maternal beta diversity (NMDS1)	0.18	55	0	0
	Maternal beta diversity (NMDS2)	0.76	55	0	0
	Morning salivary cortisol	0.99	57	0	0
	Parity	0.21	59	0	0

† Significant result after using the FDR cut-off from main analyses, corresponding to a p-value of 0.0209. 1=yes. 2=no

*** Significant result in main analyses after FDR as well as after sensitivity analysis. 1=yes. 2=no**
